# Use of speckle tracking echocardiography in evaluating cardiac dysfunction in patients with acromegaly: an update

**DOI:** 10.3389/fendo.2023.1260842

**Published:** 2023-10-20

**Authors:** Rong Huang, Jiewen Jin, Pengyuan Zhang, Kemin Yan, Hanrong Zhang, Xin Chen, Wei He, Hongyu Guan, Zhihong Liao, Haipeng Xiao, Yanbing Li, Hai Li

**Affiliations:** ^1^ Department of Endocrinology, The First Affiliated Hospital, Sun Yat-Sen University, Guangzhou, China; ^2^ Department of Medical Ultrasonics, The First Affiliated Hospital, Sun Yat-Sen University, Guangzhou, China

**Keywords:** acromegaly, cardiac dysfunction, speckle tracking echocardiography, myocardial strain, diagnosis

## Abstract

In recent years, cardiovascular disease has garnered increasing attention as the second leading cause of death in individuals with acromegaly, following malignancy. Identifying cardiac dysfunction early in acromegaly patients for timely intervention has become a focal point of clinical research. Speckle tracking echocardiography, a well-established ultrasound technique, surpasses conventional Doppler ultrasound in its sensitivity to assess both local and global cardiac mechanics. It can accurately detect subclinical and clinical myocardial dysfunction, including myocardial ischemia, ventricular hypertrophy, and valvular changes. Over the past five years, the use of speckle tracking echocardiography in acromegaly patients has emerged as a novel approach. Throughout the cardiac cycle, speckle tracking echocardiography offers a sensitive evaluation of the global and regional myocardial condition by quantifying the motion of myocardial fibres in distinct segments. It achieves this independently of variations in ultrasound angle and distance, effectively simulating the deformation of individual ventricles across different spatial planes. This approach provides a more accurate description of changes in cardiac strain parameters. Importantly, even in the subclinical stage when ejection fraction remains normal, the strain parameters assessed by speckle tracking echocardiography hold a good predictive value for the risk of cardiovascular death and hospitalization in acromegaly patients with concomitant cardiovascular disease. This information aids in determining the optimal timing for interventional therapy, offering important insights for cardiac risk stratification and prognosis. In the present study, we comprehensively reviewed the research progress of speckle tracking echocardiography in evaluating of cardiac dysfunction in acromegaly patients, to pave the way for early diagnosis of acromegaly cardiomyopathy.

## 1 Introduction

Acromegaly is a chronic neuroendocrine disorder primarily attributed to pituitary neuroendocrine tumors (PitNETs) secreting growth hormone (GH), thereby prompting excessive production of insulin-like growth factor 1 (IGF-1). It is characterized by facial roughness and enlarged extremities due to excessive soft tissue growth and also associated with bone and joint lesions and related metabolic syndrome (1–3). The incidence of acromegaly exhibits variability, ranging between 83 and 133 cases per million individuals, according to recent research (4–10). Long-term exposure to elevated GH and IGF-1 concentrations results in higher mortality rates primarily attributed to cardiovascular, cerebrovascular, and pulmonary dysfunction, leading to a 30% notable reduction in life expectancy (2, 11). Over the past decade, cardiovascular complications have become one of the leading causes of mortality in acromegaly patients. Although the prevalence of cardiovascular disease in acromegaly patients has decreased from 44% to 23% in recent years, it still stands as a significant cause of death in this population, second only to malignant tumors (12).

These cardiovascular complications include acromegalic cardiomyopathy, hypertension, arrhythmias, and valvular disease (13). Acromegalic cardiomyopathy is characterized by left ventricular hypertrophy (LVH) and diastolic dysfunction, with a prevalence ranging from 11% to 78% (mean 41.9%) (14). The prevalence rates for hypertension, arrhythmias, and valvular disease stand at 11.9%–54.7% (mean 33.6%), 89%, and 75%, respectively, with arrhythmias and valvular disease often remaining asymptomatic (13, 15). Patients with acromegaly suffering from cardiovascular disease exhibit a twofold increase in mortality compared to those without such complications (16). GH can enhance the sensitivity and content of myofilament calcium, L-type calcium channels, and collagen deposition, thereby regulating the growth and metabolism of cardiomyocytes. In contrast, IGF-1 can reduce cardiomyocyte apoptosis, preventing the loss of these cells and contributing to the maintenance of cardiac function. However, when the heart is exposed to high concentrations of GH and IGF-1, it undergoes morphological and functional adaptive changes, primarily attributable to two mechanisms: 1) the direct toxic effects of excessive GH and IGF-1 on the heart; 2) the induction of arterial hypertension and disruption of glucose and lipid metabolism. Myocardial damage in acromegaly progresses through distinct stages: 1) asymptomatic left ventricular hypertrophy and increased systolic function in the early stage, 2) obvious LVH, diastolic dysfunction and decreased systolic function in the middle stage, 3) in the end stage, it develops into dilated cardiomyopathy, which can ultimately result in heart failure. Importantly, myocardial damage in the early stage is potentially reversible (17, 18). Therefore, the early evaluation and diagnosis of cardiovascular disease in acromegaly remain a major clinical concern.

Echocardiography presents a readily accessible technique for evaluating both structural and functional cardiac abnormalities (19). However, due to individual heterogeneity, traditional two-dimensional echocardiography indices of myocardial systolic-diastolic function exhibit low sensitivity, leading to a high rate of missed diagnosis for subclinical myocardial function impariments (20). Consequently, there is a pressing need for a non-invasive diagnostic tool characterized by speed, repeatability, and high resolution to enhance clinical diagnosis and treatment.

Speckle tracking echocardiography (STE) emerges as a relatively recent ultrasound technique that allows quantitative analysis of both regional and overall myocardial motion and deformation. Importantly, it overcomes the well-documented limitations of Doppler ultrasound, including angle dependence, susceptibility to noise interference, and interobserver variability (21, 22). Currently, STE finds prominent clinical applications in: 1) evaluating subclinical myocardial dysfunction, such as cardiomyopathy resulting from various causes, viral myocarditis, and heart failure with preserved ejection fraction, etc. 2) distinguishing between types of cardiac wall hypertrophy, such as hypertrophic cardiomyopathy and myocardial amyloidosis, (3) diagnosing ischemic heart diseases, such as coronary heart disease, 4) evaluating cardiac function in cancer patients, including monitoring cardiotoxicity induced by chemotherapeutic agents (23). This ultrasound technique has proven invaluable in the clinical diagnosis and prognostic stratification of hypertrophic cardiomyopathy, pericardial diseases, and aortic regurgitation (24–26). For example, STE enables the prediction of the optimal timing to initiate antihypertensive therapy in patients with class I hypertension (AH) and determines the most suitable time for intervention in patients with asymptomatic severe heart valve disease. Notably, data indicates improvements in global longitudinal strain (GLS) following 24 weeks of hypertension medication, even if AH has not returned to normal levels. In patients with atrial fibrillation, peak left atrial longitudinal strain (PALS) emerges as an independent predictor of recurrence following conversion to sinus rhythm, exhibiting an 85% sensitivity and 99% specificity. When evaluating diastolic function in heart failure patients, PALS demonstrates superior feasibility compared to atrial volumes, resulting in a 75% reduction in the diagnosis of diastolic function of uncertain clinical significance and significantly improving clinical diagnostic accuracy (27). In the field of PitNETs, STE has been successively used in Cushing’s disease (28), thyrotropin-secreting tumors (29, 30), and acromegaly, with the latter being the most extensively studied. STE has emerged as a crucial tool in evaluating cardiac function among acromegaly patients. Its application in acromegaly represents a novel clinical practice, offering a new avenue and reference for the diagnosis and treatment of acromegaly. Therefore, in the present study, we comprehensively review cardiac dysfunction detected through speckle tracking technology in patients with acromegaly. Additionally, we summarize the ultrasound characteristics indicative of subclinical myocardial functional injuries. These findings may open up new perspectives to guide future clinical management.

## 2 Speckle tracking ultrasound technology

Speckle tracking ultrasound technology and traditional two- or three-dimensional digital echocardiography images, employ image processing algorithms to identify small and stable myocardial footprints or spots generated by the interaction between ultrasound waves and myocardial tissue in the selected region of interest. Tracking the distance between frames and spots or their spatial-temporal displacement (regional strain velocity vector) in each cardiac cycle, provides valuable information on focal, phased, and global myocardial strain (23). The conventional method for measuring Lagrangian strain was originally developed using tissue Doppler (31). However, its precision and usefulness are limited due to its angular dependence and sensitivity to noise. STE effectively addresses these limitations and can accurately distinguish between normal myocardial segmental displacements and those occurring passively due to myocardial hypertrophy or the restriction of adjacent myocardial tissue (23). Two-dimensional STE (2D-STE) assesses global circumferential strain (GCS) and global radial strain (GRS) through a combination of three short-axis views and three apical views to measure GLS. In contrast, three-dimensional STE (3D-STE) requires only one apical image acquisition to automatically measure GCS, GRS, and GLS. While 2D-STE involves acquiring multiple 2D images over multiple cardiac cycles, making it more time-consuming, it offers higher temporal and spatial resolution compared to the single full-volume acquisition of 3D-STE (32). These strain indicators in STE represent the ratio of the maximum contraction change in myocardial length in all directions to its initial size. During systole, when the myocardium contracts, the length decreases, resulting in strain parameters typically expressed as negative values. Lower negative values indicate better ventricular systolic function.

## 3 2D-STE

2D-STE is an innovative ultrasound technique that combines speckle tracking with two-dimensional ultrasound to assess longitudinal strain (LS), radial strain (RS), and circumferential strain(CS) associated with myocardial contractility that can occur at the myocardial level. This is in contrast to the left ventricular ejection fraction (LVEF), which is commonly used clinically to evaluate left ventricular systolic function but is less sensitive to subtle ultrastructural changes (21, 33–35). Myocardial strain refers to the percentage change in myocardial length relative to its initial myocardial length in a non-stress state, enabling the direct visualization of myocardial changes such as elongation, shortening, thickening, or thinning (36). GLS is considered the most valuable parameter for clinical diagnosis and prognosis (35, 37, 38). It is frequently used in clinical practice for assessing myocardial function, stratification of disease prognosis, and defining drug threshold (39). Moreover, 2D-STE is free from defects like noise interference, angle dependence, or heterogeneity among different operators, making it highly valuable in clinical applications (40).

### 3.1 Left ventricular (LV) strain imaging

The use of 2D STE in patients with acromegaly is on the rise, with a primary focus on GLS as a key parameter for assessing changes in ventricular anatomical volume changes and kinematic alterations. Left ventricular function has always been a focal point of clinical research, thus warranting early exploration. Volschan ICM et al. were among the first to apply STE to assess cardiac function in acromegaly patients (40). Nevertheless, they observed no difference in GLS between acromegaly patients and healthy controls, matched for sex, age, AH, and diabetes mellitus(DM). This finding contradicted previous results obtained using Doppler ultrasound strain imaging (41–44). Subsequently, Popielarz-Grygalewicz et al. used 2D STE to assess GLS in patients with naive acromegaly who exhibited normal LVEF (45). Interestingly, this study revealed that the acromegaly group exhibited significantly worse GLS values when compared to the control group (-16.6% vs. -20.7%, p < 0.01). Concurrently, the study by Uzibło-Życzkowska et al. arrived at similar results as Popielarz-Grygalewicz et al. (-18.1% vs. -19.4%, p = 0.023) (46). Conversely, Gadelha P et al. obtained results similar to those of Volschan ICM et al. in their latest study (19). We hypothesize that the disparity in results may be attributed to differing inclusion criteria. Volschan ICM et al. and Gadelha P et al. included subjects who had already undergone surgery or recived somatostatin analogs(SSA) treatment, while Popielarz-Grygalewicz et al. focused on patients who had not received any treatment. As a result, Popielarz-Grygalewicz A. et al. further investigated GLS differences before and after acromegaly treatment. They found that GLS significantly improved in the group receiving appropriate treatment(using SSA for 3 months) compared to the untreated group (-20.4% vs. -20.0%, p = 0.045). However, there was no significant change in GLS after SSA treatment lasting more than 6 months. Additionally, they discovered a positive correlation between baseline GLS with BMI (r=0.446, p=0.011) as well as BSA (r=0.411, p=0.019), explaining the better GLS values in female patients, possibly related to lower BMI and BSA values in women (47).

### 3.2 Left atrial (LA) strain imaging

Only a limited number of studies have employed 2D STE to assess LA function in acromegaly patients. Uzibło-Życzkowska et al. used 2D STE to evaluate changes in LA and LV function parameters among acromegaly patients, and their findings indicated that GLS was significantly worse in these patients (46). This observation was related to the stage of the disease and the GH levels of the patients. Similarly, Koca et al. used 2D STE to assess the functional parameters of LA and LV in individuals with active, long-term acromegaly. Their research revealed a significant deterioration in the GLS of both LA and LV (48), aligning with the results of Uzibło-Życzkowska et al. Furthermore, Koca et al. were the first to identify a strong positive correlation between the extent of GLS-LA reduction in and IGF-1 levels. However, the relationship between IGF-1 and GLS-LV remained controversial (19, 40, 48).

The LA serves multiple roles within the cardiac system, including acting as a cardiac reservoir, catheter, and booster pump (49). It also plays a pivotal predictive role in major cardiovascular and cerebrovascular adverse events (MACE), such as heart failure, arrhythmias, and stroke (50–52). Given its importance in maintaining normal cardiac function, LA strain parameters can be classified into distinct categories based on their role during different phases of the cardiac cycle. These categories include left atrial reservoir strain (LASr), left atrial conduit strain (LASc), and left atrial contractile strain (LASct), with LASr receiving particular attention in clinical research. During ventricular systole, LA deformation depends on atrial blood filling and the traction exerted by the mitral annulus due to LV contraction. Consequently, LASr can serve as an indicator of LA myocardial fibrosis and LV functional characteristics, facilitating assessments of LV dysfunction classification and the risk of recurring MACE (e.g., atrial fibrillation, heart failure, etc.) (53, 54). Nevertheless, it is worth noting that LASr’s clinical information pertaining to the diastolic phase may be limited, and further clinical studies are warranted to explore the predictive potential of LASc and LASct to address this limitation. Routine LA monitoring and evaluation in clinical diagnosis and treatment could help identify subclinical risk events promptly. **Table 1** summarizes the application of 2D STE with acromegaly patients.

**Table 1:** Application of 2D STE in patients with acromegaly.

Year	First author	Patients included	Disease state	Cardiac region	Strain Parameters			
					Improved	Worse		Normal
2017	Volschan ICM (40).	37	Naive : Persistent non-remission: Remission (14:16:7)	LV	—	—		GLS
2020	Uzibło-Życzkowska B (46).	30	NA	LA, LV	—	GLS		—
2020	Popielarz-Grygalewicz A (45).	43	Naive	LV	—	GLS		—
2022	Gadelha P (19).	25	Remission : Naive (20:5)	LV	—	GRS		GLS, GCS, LVT
2022	Koca H (48).	50	Recurrence : Persistent non-remission(45:5)	LA, LV	—	GLS		—
2023	Popielarz-Grygalewicz A (47).	35	Naive	LV	—	—		GLS

LV, left ventricular; LA, left atrial; GLS, global longitudinal strain; GCS, global circumferential strain; GRS, global radial strain; LVT, left ventricular twist; NA, not available.

The symbol "—" means “NA, not available”.

## 4 3D-STE

Compared to 2D STE, the analysis of cardiac functional parameters using 3D STE offers several advantages. It is faster, demands less technical expertise, reduces interobserver variability, and enhances reproducibility (55). Furthermore, 3D STE enables the calculation of area strain (AS) by measuring wall strain in the circumferential direction (56). Despite some inherent drawbacks, such as lower temporal and spatial resolution and a tendency to underestimate rotation and distortion, the benefits of 3D STE in measuring cardiac strain parameters outweigh these limitations. Simultaneously, there is also a “stitching noise” observed between individual subvolumes (57, 58). However, it is essential to note that the advantages of employing 3D STE for measuring cardiac strain parameters significantly outweigh these disadvantages. Over the years, there has been an increasing trend toward the adoption of 3D STE for the assessment of cardiac dysfunction in patients with acromegaly.

### 4.1 Left ventricular (LV) strain imaging

In 2018, Kormányos et al. conducted pioneering research using 3D STE to investigate LV rotation patterns and mechanical changes in acromegaly patients. Their findings revealed significant alterations in LV basal, apical, and ventricular rotation parameters among patients with acromegaly (59). Notably, only torsion time displayed a significant difference between the active and inactive acromegaly subgroups. Parameters associated with LV rotational mechanics offer valuable insights into cardiac injury beyond what can be gleaned from LVEF. They provide a means to predict myocardial recovery clinically and offer indicators of parasympathetic autonomic function (60). Nevertheless, studies on myocardial rotational mechanics in acromegaly remain limited. While 3D STE can simultaneously measure cardiac volume, strain, and rotation parameters, no research has yet employed 3D STE to comprehensively assess all relevant cardiac parameters in acromegaly patients, offering a complete assessment of cardiac injury and prognosis.

Kormányos et al. used 3D STE to evaluate cardiac radial strain (RS), longitudinal strain (LS), circumferential strain (CS), and three-dimensional comprehensive strain (3DS) in active acromegaly patients (61). Their findings indicated that GRS was significantly improved in patients with active acromegaly compared to controls, suggesting increased myocardial strain and contractility. Only CS displayed statistically significant differences between the active and inactive acromegaly subgroups, consistent with subsequent observations by Gadelha P et al. (19) Furthermore, Gadelha P’s team identified a reduction in GRS in patients with inactive acromegaly, although it remained within the normal range (35%–39%) (62). Interestingly, in previous studies on LV rotation and torsional mechanics conducted by Kormányos’ team, both LV rotation parameters exhibited some degree of reduction, suggesting myocardial injury, regardless of acromegaly activity (59). This appears to contradict the observed increase in GRS. However, it is reasonable to speculate whether there may exist a self-regulatory threshold within the LV, whereby myocardial rotational parameters decrease while GRS improves compensatorily to ensure normal cardiac function. When the disease is effectively controlled, GRS returns to normal range, indicating reversibility and highlighting the role of disease control in mitigating myocardial damage. GRS is inherently more variable than GLS, with small changes in specific regions capable of yielding significant GRS results (62, 63). Consequently, when assessing myocardial GRS changes, a comprehensive analysis of both segmental and global changes should be considered.

To date, no comprehensive study has explored the factors influencing LV deformation using 3D STE. Nemes A et al. attempted to use 3D STE to assess the effect of DM on LV rotation and deformation parameters in acromegaly patients (64). Their study found that, whether with or without diabetes, LV tip torsion parameters exhibited a certain degree of reduction in acromegaly patients. However, an improvement in GRS was observed only in the acromegaly group without diabetes, with GRS levels in the acromegaly group with diabetes resembling those in the control group. As discussed earlier, better GRS may manifest as a compensatory response to cardiac damage, which may be offset by the onset of DM. Previous studies have suggested that DM can accelerate aortic sclerosis in patients with acromegaly (65). Consequently, it is reasonable to speculate that DM may exacerbate myocardial deformation in acromegaly patients and expedite the progression of myocardial injury. Further clinical investigations are warranted to determine whether hypertension and dyslipidemia yield similar outcomes in acromegaly patients.

### 4.2 Left atrial (LA) strain imaging

The LA functions as an auxiliary pump for the ventricle, undergoing physiological remodeling to support the ventricle’s ejection function during increased load. However, the magnitude of atrial enlargement is limited, and once pathological remodeling, such as fibrosis or atrial fibrillation occurs, reversal becomes unlikely. Thus, timely recognition and intervention for left atrial injury are of paramount importance (66). Kormányos et al. applied 3D STE to evaluate the volume and functional parameters of the LA in acromegaly patients (61). Their investigation revealed that volume parameters (maximum end-systolic volume, pre-systolic atrial volume, and end-diastolic minimum volume) and strain parameters (RS and 3DS) were significantly more favorable in acromegaly patients compared to healthy controls. However, GCS experienced a moderate deterioration. Particularly, the improvement in GRS was more pronounced in the subgroup with active acromegaly, mirroring findings observed in the left ventricle. Kormányos et al. suggested that this LA remodeling might represent a compensatory mechanism in response to diastolic filling impairment in acromegaly patients. LV remodeling typically progresses through stages, with early manifestations of myocardial hypertrophy and increased myocardial contractility. This is followed by the development of end-diastolic underfilling, culminating in decreased LVEF, irreversible ventricular remodeling, and congestive heart failure at the end stage (67).

Popielarz-Grygalewicz A et al. indicated that nearly 80% of acromegaly patients exhibit increased LAVi, whereas LVH is observed in less than 50% of patients. This suggests that adaptive LA remodeling takes precedence over LV remodeling in the cardiac progression of acromegaly patients. Furthermore, the study identified a negative correlation between LAVi and GLS, highlighting that the LA, functioning as a cardiac reservoir, catheter, and booster pump, shares a Frank-Starling regulatory mechanism similar to the LV. The LA and LV work in dynamic cooperation (47). Consequently, periodic STE assessments of the LV and LA can track the progression of cardiac damage in acromegaly patients.

### 4.3 Right atrial (RA) strain imaging

Limited research has investigated the functional parameters of RA in patients with acromegaly. Historically, the role of the RA has been characterized as ‘the first to live and the last to die, underscoring its pivotal role in maintaining normal blood supply to the heart. Using 3D STE for a quantitative assessment of RA function can greatly enhance our understanding of its function and remodeling (68). Kormányos et al. used 3D STE to assess the RA in acromegaly patients (69). Their findings revealed a significant increase in volume parameters (maximum end-systolic volume, pre-systolic atrial volume, end-diastolic minimum volume) and an improvement in strain parameters (RS and 3DS). Conversely, GCS and GLS exhibited some degree of deterioration, which is consistent with the results of left atrial evaluations. Moreover, this study revealed that myocardial remodeling can be reversed upon the control of IGF-1, as observed in LA. However, further experimentation is warranted to explore whether the evolution of the RA parallels that of the LA.

### 4.4 Mitral annulus (MA) imaging

The prevalence of heart valve disease in acromegaly patients approaches 75% (13), with most cases involving asymptomatic valvular changes. The mitral valve is predominantly affected, followed by the aortic valve, typically manifesting as mitral regurgitation (15). Cardiac valve injury and myocardial hypertrophy follow distinct disease progression mechanisms, and to some extent, myocardial hypertrophy can be improved with intervention. Conversely, valvular injury represents an irreversible process, with the risk of valvular disease increasing by 19% annually as the disease advances (70). Therefore, early identification of acromegaly is very critical. Conventional two-dimensional Doppler ultrasound often underestimates the diameter of the MA, whereas experimentally evidence underscores the superior accuracy of three-dimensional ultrasound compared to the two-dimensional ultrasound, accurately reflecting the real shape of the valvular annulus (71–73). Nemes A et al. endeavored to evaluate the MA of acromegaly patients using 3D STE and observed a significant increase in its diameter, area, and perimeter compared to the control group (74). Nonetheless, the functional parameters of the MA exhibited no significant changes, which explains why acromegaly patients often show asymptomatic valve injuries. The activity of both LA and LV can influence MA contraction (75). We postulate that MA dilatation in acromegaly patients may mirror the compensatory mechanism observed in GRS (19, 61, 76). Mitral annulus dilatation could potentially serve as a regulatory mechanism against cardiac injury caused by persistently elevated concentrations of GH and IGF-1. Nonetheless, there is a dearth of research elucidating the mechanism behind mitral annular injury, warranting further exploration. **Table 2** summarizes the application of 3D STE in acromegaly patients.

**Table 2:** Application of 3D STE in patients with acromegaly.

Year	First author	Patients included	Disease state	Cardiac region	Strain Parameters		
					Improved	Worse	Normal
2017	Kormányos Á (59).	20	Persistentnon-remission:Remission(12:8)	LV	—	LVBR, LVT, LVAR	—
2018	Kormányos Á (61).	19	Persistentnon-remission:Remission(11:8)	LA	GRS, 3DS	GLS	GCS, GAS
2020	Kormányos Á (69).	22	Persistentnon-remission:Remission(10:12)	RA	3DS	—	GLS, GRS, GCS, GAS
2020	Kormányos Á (76).	25	Persistentnon-remission:Remission(14:11)	LV	GRS	—	GLS, GAS, GCS, 3DS
2021	Nemes A (74).	24	Persistentnon-remission:Remission(12:12)	MA	—	—	—
2021	Nemes A (64).	24	Persistentnon-remission:Remission(15:9)	LV	GRS	LVAR, LVT	GCS, GLS, GAS, 3DS, LVBR

LV, left ventricular; LA, left atrial; RA, right atrial; MA, Mitral annulus; GLS, global longitudinal strain; GCS, global circumferential strain; GRS, global radial strain; GAS, global area strain; LVT, left ventricular twist; LVBR, left ventricular basal rotation; LVAR, left ventricular apical rotation; 3DS, three-dimensional comprehensive strain.

The symbol "—" means “NA, not available”.

## 5 Perspectives on the use of STE in assessing the effects of acromegaly treatment

The primary treatments for acromegaly include surgery, drugs, and radiotherapy. Timely surgery serves as the cornerstone of early treatment, while drugs and radiotherapy are commonly employed as second-and third-line options when clinical remission is not achieved following surgical treatment. However, even after reaching the clinical remission target for GH, additional treatment for complications remains necessary (3). In general, prompt treatment can partially reverse early myocardial damage. Presently, the primary objective in managing acromegaly is to maintain GH and IGF-1 secretion at normal levels, thereby decelerating disease progression and reducing mortality (14). Previous studies have demonstrated the significant advantages of SSA in acromegaly patients, particularly in terms of cardiac electrophysiology. They lead to reductions in heart rate, ventricular extrasystoles, and QT interval dispersion, thus reducing the incidence of arrhythmias (77). Additionally, SSA treatment can mitigate LVH, and restore normal systolic and diastolic functions (18). CMRI(cardiac magnetic resonance imaging) has been employed to assess changes in LV function and structure before and after drug treatment in acromegaly. Results have shown significant improvements in LVEF, LVH, left ventricular end-systolic volume, and end-diastolic volume (78–80), warranting further exploration in future research. It is worth discussing whether other atria and ventricles in acromegaly undergo similar structural and functional changes and whether more sensitive indicators can predict heart injury. Speckle tracking ultrasound, especially 3D-STE, can provide insights into myocardial torsional, GRS, GCS, and GLS. This allows for the early detection of myocardial functional impairment before a decrease in LVEF evident. Furthermore, it can distinguish between pathological myocardial abnormalities and physiologically adaptive changes based on characteristic strain patterns. It can also detect the reduction in pathological diastolic function caused by myocardial hypertrophy before the decrease in LVEF, facilitating timely intervention to reverse myocardial injury. While the cardiac strain parameters of acromegaly patients tend to improve to some extent after treatment, a more extended follow-up study is needed to ascertain the predictive value of STE in cardiac function recovery.

## 6 Conclusion

The STE can identify the changes of early atrioventricular motion pattern changes and evaluate myocardial strain parameters, offering valuable evidence of subclinical myocardial injury. This information is crucial for the clinical management and prognosis of cardiac dysfunction in acromegaly patients. However, since STE is a relatively recent technique, current research on its application to explore myocardial strain parameters in acromegaly patients is insufficient and long-term studies evaluating the predictive value of strain parameters are lacking. Further research is required to determine the accuracy of strain parameters in predicting the prognosis and survival outcomes of acromegaly patients. Nonetheless, we can affirm that the speckle tracking technique holds significant potential application prospects in acromegaly and cardiac dysfunction, representing a breakthrough in diagnosis. In clinical practice, the selection of imaging techniques depends on the indications and information requirements for a given case. Often, a combination of one or more imaging modalities is employed to achieve an accurate disease diagnosis. Commonly used cardiac imaging techniques for evaluating myocardial performance include echocardiography, CMRI, ventriculography, myocardial radionuclide imaging, and CT(Computed Tomography) (**Table 3**). While these techniques generally exhibit good correlation, each possesses distinct advantages and limitations (20, 59, 81–87), which can contribute to larger standard deviations in myocardial function indices. Consequently, clinical diagnosis and treatment necessitate reasonable selection and the exploitation of complementary advantages. In the future, it is anticipated that STE will be used alongside other cardiac imaging techniques for the clinical diagnosis and prognosis of acromegaly patients.

**Table 3:** Different imaging techniques for assessing myocardial function.

	Advantages	Limitations
Conventional echocardiography (20, 81)	• Most used, clinical first choice • Convenient and quick • Safe and cheap	• Apical fluoroscopy shortening • Highly dependent on geometric assumptions • The sensitivity of myocardial systolic-diastolic function indicators is low, resulting in a high rate of missed diagnosis of subclinical myocardial dysfunction
STE (59, 82, 83)	• Non-invasive and radiation-free • High speed, high accuracy and good repeatability • Incremental value independent of EF • Not restricted by geometry • Without apical fluoroscopy shortening • Global and regional ventricular function can be evaluated.	• Poor temporal and spatial resolution • High requirements for image quality • Tends to underestimate the degree of rotation and distortion of LV, and there may be “splicing noise” • Limited by poor endocardial depiction, resulting in underestimation of strain parameters. • With inter-observer heterogeneity
CMRI (84, 85)	• Radiation-free • High temporal and spatial resolution, high accuracy and repeatability • High signal-to-background ratio, easy to determine the intimal boundary; • Three-dimensional tomography can be obtained in any plane direction, which is not affected by the patient’s body shape. • Comprehensive evaluation of cardiac anatomy and function • The gold standard to determine myocardial function	• Expensive and requires highly skilled operators • Time-consuming • Not suitable for pacemaker/defibrillator patients/claustrophobic patients • Movement during image acquisition can cause artifacts
RVG (85)	• High repeatability and Low operator dependence • Independent of geometric assumptions • Accurate determination of EF, facilitating cardiac evaluation in patients with ischemic cardiomyopathy, multiple wall motion abnormalities, and altered left ventricular geometry	• With radiation exposure • Unable to assess localized systolic thickening • Unable to get anatomical information incidentally • Low sensitivity to ventricular hypertrophy
X-ray ventriculography (86)	• High speed and high accuracy • High signal-background noise ratio, good boundary resolution • Real-time performance	• Expensive • Invasive and x-ray exposure; • Single-plane imaging, depending on geometric assumptions • Risk of premature ventricular contractions;
CT (87)	• Non-invasive • high signal-to-background ratio, easy to determine the intimal boundary • Simultaneous analysis and study of the relationship between coronary artery disease and cardiac function	• X-ray exposure • Poor temporal resolution • Require injection of contrast agent, causing kidney damage • Limited by arrhythmias
CCTA (86)	• Non-invasive • High temporal and spatial resolution • Left ventricular ejection fraction and wall motion can be accurately evaluated, and the accuracy is better than that of ventriculography and ultrasound.	• Iodide contrast agent and radiation exposure • Artifacts and poor image quality will occur in the case of fast heart rate.

CMRI, cardiac magnetic resonance imaging; STE, speckle tracking echocardiography; RVG, radionuclide ventriculography; CCTA, cardiac Computed Tomographic Angiography;CT, Computed Tomography.

## 7 Limitation

This paper is a summary based on our own understanding of the literature and although we have tried to be as objective as possible in our analysis, we still cannot exclude a strong subjectivity. Also, this paper is only a summary of the application of STE in the acromegaly population, and the scope of discussion is narrow, so the results should not be interpolated to the general population, pending further expansion of the study population for comparative analysis. As STE has only begun to show its clinical application in the last decade, the number of relevant papers included in this article is limited, and more relevant studies are expected in the future to further explore the diagnostic value of STE in cardiac dysfunction in the acromegaly population.

## Author contributions

HL: Supervision, Writing – review & editing. RH: Data curation, Formal Analysis, Writing – original draft. JJ: Data curation, Formal Analysis, Writing – original draft. PZ: Data curation, Formal Analysis, Writing – original draft. KY: Methodology, Writing – review & editing. HZ: Investigation, Writing – review & editing. XC: Methodology, Writing – review & editing. WH: Methodology, Software, Writing – review & editing. HG: Methodology, Writing – review & editing. ZL: Supervision, Writing – review & editing. HX: Supervision, Writing – review & editing. YL: Supervision, Writing – review & editing.
